# National Yearly Trend of Utilization and Procedural Complication of the Watchman Device in the United States

**DOI:** 10.7759/cureus.25567

**Published:** 2022-06-01

**Authors:** Biraj Shrestha, Bidhya Poudel, DilliRam Poudel, Julian Diaz Fraga

**Affiliations:** 1 Internal Medicine, Reading Hospital Tower Health, Reading, USA; 2 Internal Medicine, AMITA Health Saint Francis Hospital, Evanston, USA; 3 Rheumatology, Indiana Regional Medical Center, Indiana, USA; 4 Cardiology, Reading Hospital Tower Health, Wyomissing, USA

**Keywords:** factual, databases, perioperative complication, percutaneous closure, left atrial appendage occlusion, atrial fibrillation

## Abstract

Background

Complication from the Watchman device (Boston Scientific Corp, Marlborough, Massachusetts) is operator-dependent, with the latest EWOLUTION trial showing low complication rates (1.8%) thought to be due to maturing physician experience.

Objectives

The objective of this study is to understand the yearly trend of utilization and complication rates of the Watchman device in hospitalized patients.

Methods

The national inpatient sample (NIS) was queried for all hospitalization with primary atrial fibrillation or flutter from 2016 to 2019 with percutaneous left atrial appendage occlusion (LAAO). The frequency of peri-procedural complications, including death, stroke, major bleeding requiring blood transfusion, pericardial effusion, post-op hypotension, cardiac arrest, postprocedural CHF, implant displacement/leak, systemic embolism, and requiring repeat procedures, were assessed.

Results

From 2016 to 2019, an estimated 60,350 LAAO procedures were performed. The majority of the procedure was done in white (84.88%), males (58.40%), with a mean age of 76, at teaching hospitals (88.27%). Complication rates were around 5.72%, with no change from 2016 to 2019 (annual percentage change, APC: 6.23; p-value: 0.170) despite rapid increase in yearly utilization of Watchman from 1.12% in 2016 to 5.45% in 2019 (APC: 62.30; p-value of 0.013). Pericardial effusion (3.41%) was the most common complication, followed by bleeding requiring transfusion (1.40%) that had no significant change over time.

Conclusion

Our study demonstrates that trend of complications with the Watchman device implantation in the real-world practice didn’t improve over time, possibly due to characteristics inherent to the device and patient population. Hence, we expect a further drop in nationwide complication rates with the improved design of Watchman-FLX and increased placement experience.

## Introduction

Atrial fibrillation is associated with a three-fold increase in thromboembolic stroke in patients not on anticoagulation [1]. However, the physician runs into a problem when selected patients cannot be anticoagulated due to contraindications or difficulty in medication adherence. Left atrial appendage occlusion (LAAO) came as a potential solution. The Protect-AF trial demonstrated a non-inferior rate of cardiovascular death, systemic embolism, or stroke compared to warfarin alone [2]. The first FDA-approved LAAO device in the USA was the Watchman device (Boston Scientific Corp, Marlborough, Massachusetts), approved in March 2015, followed by Watchman FLX, approved in July 2021, and Abbott’s Amplatzer Amulet for Atrial Fibrillation (Abbott Laboratories, Chicago, Illinois), approved in August 2021 [3,4,5].

The incidence of complications reported post-LAAO ranges from 1.8% to 8.7%, with pericardial effusion being the most common complication with a reported range between 0.29% and 2.4% [6,7]. There has been a noted overall decrease in complication rates from the initial documented 8.7% in the Protect-AF trial to the most recent EWOLUTION trial reporting around 1.8% [8], which some of the authors believe could be explained by the maturing experience of the implanting physician [7]. Patients who were included in the study were older males with a mean age between 71.7 to 74 years, a mean CHA2DS2-VASc score range of 3.4 to 4.5, with a history of hypertension in 81.7% to 89.6%, and diabetes in 24.4 to 33.8%, Prior stroke/transient ischaemic attack (TIA) in 17.7 to 29.7 % and congestive heart failure (CHF) in 26.8 to 34.2% [6-8]. 

To better understand these trends, we queried an inpatient US national database to understand the yearly utilization, complication rates, yearly trend of complications, and the patient demographics contributing to complications. Our research questions included: a) what is the annual utilization of the LAAO device?; b) was there a demographic difference between groups with and without complications?; c) what are the complication rates and inpatient mortality of LAAO procedures?; and d) what is the yearly trend and annual percentage change of utilization of Watchman and total complication rates?

## Materials and methods

We conducted a retrospective study to assess the peri-procedural complications associated with Watchman device implantation on the National Inpatient Sample (NIS) databases from 2016 to 2019. The NIS database is a part of the Healthcare Cost and Utilization Project (HCUP). It represents a 20% stratified sample of the US hospital discharges (weighted estimate of approximately 35 million discharges annually)[9]. Each NIS database entry contains information on patient demographics, including race, age, sex, insurance status, primary and secondary procedures, hospitalization outcome, and total cost and length of stay. There has been a good correlation between results from this database and other hospitalization discharge databases in the United States [10]. This study did not involve any patient identification data; hence we did not seek an IRB approval.

**Figure 1:**
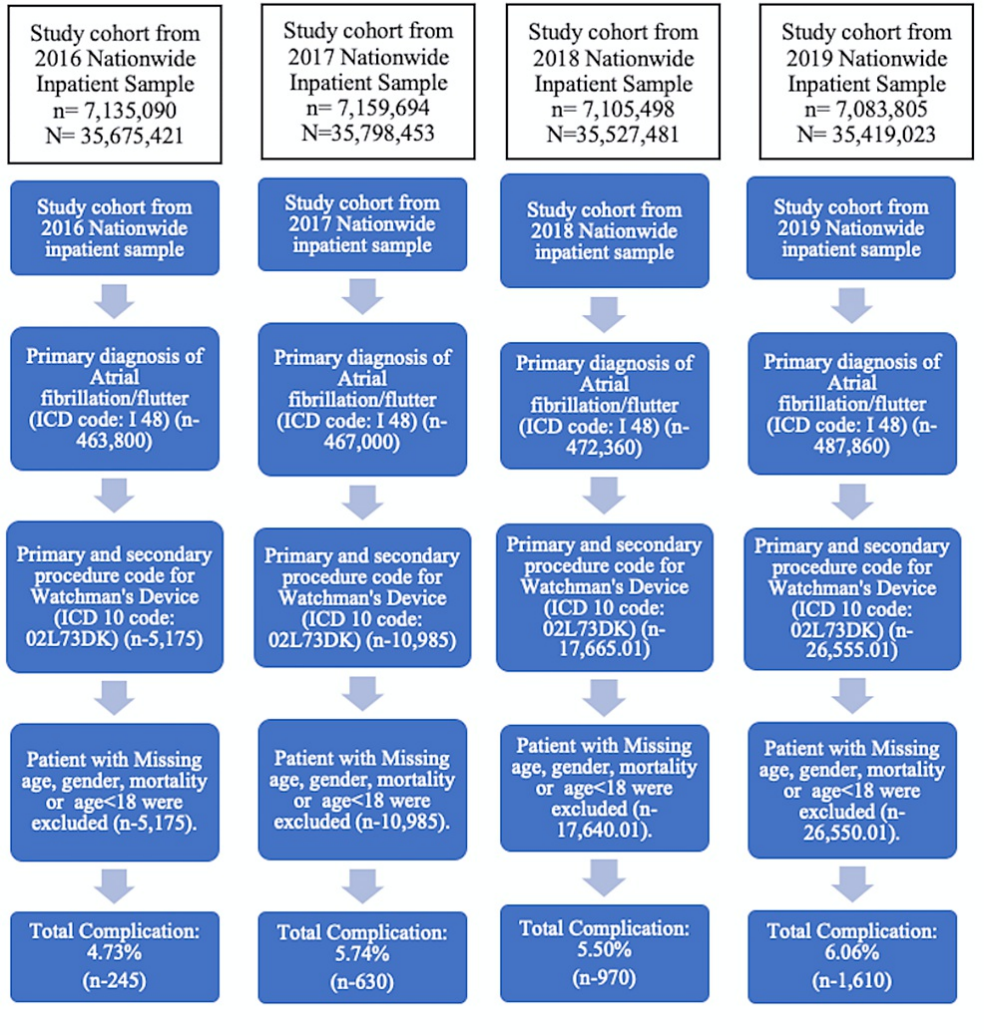
Diagram showing inclusion steps of patients using National Inpatient Sample ICD: International Classification of Diseases; N: weighted sample data; n: observed data

We identified hospitalizations with a primary diagnosis of atrial fibrillation or flutter with an International Classification of Diseases 10 (ICD 10) code diagnosis of I48. In addition, we identified Watchman implantation with procedure code 02L73DK (occlusion of left atrial appendage a with intraluminal device, percutaneous approach). We excluded hospitalization of patients aged less than 18 years and those with missing sex, age, or in-hospital mortality status (Figure 1). Our primary outcome was in-hospital mortality. Other secondary outcomes included major adverse events, including stroke, major bleeding requiring blood transfusion, pericardial effusion, post-op hypotension, pneumothorax, systemic embolism, and implant-related complication, which were assessed using ICD 10 codes in secondary positions (supplementary material in appendix Table 3) [11]. We checked individual hospitalization logs from the database for repeat procedures (removal of the intraluminal device, endoscopic LAAO, percutaneous LAAO other than Watchman, open LAAO) we found based on the ICD-10 code to ensure that the repeat procedure date occurred after the watchman procedure was done. We divided observations into any complication group and no complication group. We then described patient-level characteristics of two groups, such as age, sex, race, median household income according to the zip code, primary payer (federal versus private insurance), hospital-level characteristics such as hospital location (urban versus rural), hospital bed size (small versus medium versus large), hospital region (North East, Mid-West/ Northcentral, South, and West), and hospital teaching status (non-teaching versus teaching). We used ICD 10 codes to identify specific comorbidities, including obesity, hypertension, diabetes, coronary artery disease, chronic kidney disease (CKD) stage, coronary artery bypass grafting (CABG) history, hyperthyroidism, alcohol use disorder, mitral valve stenosis, stroke, peripheral vascular disease, and anemia (supplementary material in appendix Table 4). CHA2DS2-VASc was calculated using appropriate ICD-10 in the secondary diagnosis field as per supplemental material online (supplementary material in appendix Table 5). We also captured the comorbidity burden with the Elixhauser Comorbidity Index. The cost to charge ratio provided by Healthcare Cost and Utilization Project (HCUP) was used to calculate hospital stay costs. Finally, we calculated the yearly utilization rate of watchman procedure and complication rate from 2016 to 2019 and described the trend using joinpoint.

Statistical analysis

The statistical software package STATA version 14.2 (StataCorp LLC, College Station, Texas) was used to perform statistical calculations. Continuous variables were presented as mean (SD), and categorical variables were expressed as percentages. Weighted estimations of hospitalization-level data were generated to produce a representative estimate of the entire US population of hospitalized patients. Differences between continuous variables were tested using the student's t-test, and Chi-square statistics were reported for categorical variables. We considered p-values of less than 0.05 as statistically significant. We accommodated the complex survey design of the database using svyset and svy functions in STATA. We also conducted a joinpoint regression analysis to observe the trend of watchman incidence/complication over included years using Joinpoint Trend Analysis Software version 4.9.0.0 (supplied publicly under Surveillance Research Program by National Cancer Institute). Finally, we used Statistical Analysis Software (SAS) version 9.4 (SAS Institute Inc., Cary, North Carolina) to compute the Elixhauser Comorbidity Index, which uses 38 comorbidity measures to calculate the risk of in-hospital mortality and 30-day all-cause readmissions.

## Results

**Figure 2:**
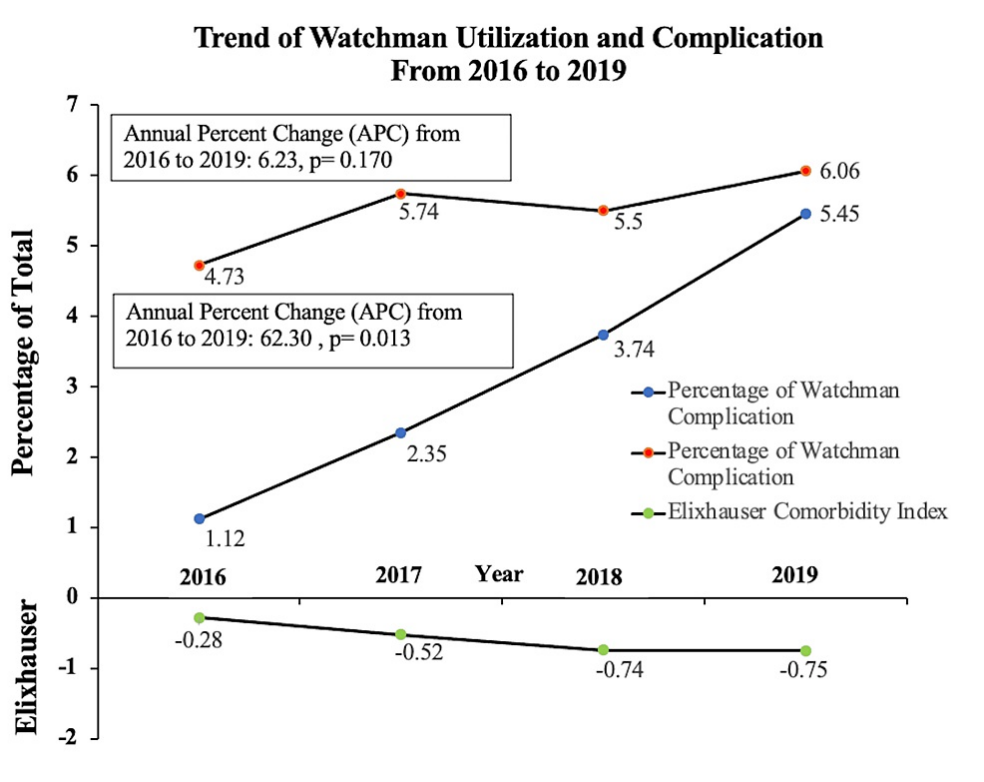
Diagram showing yearly trend of Watchman utilization and complication from 2016 to 2019

**Figure 3:**
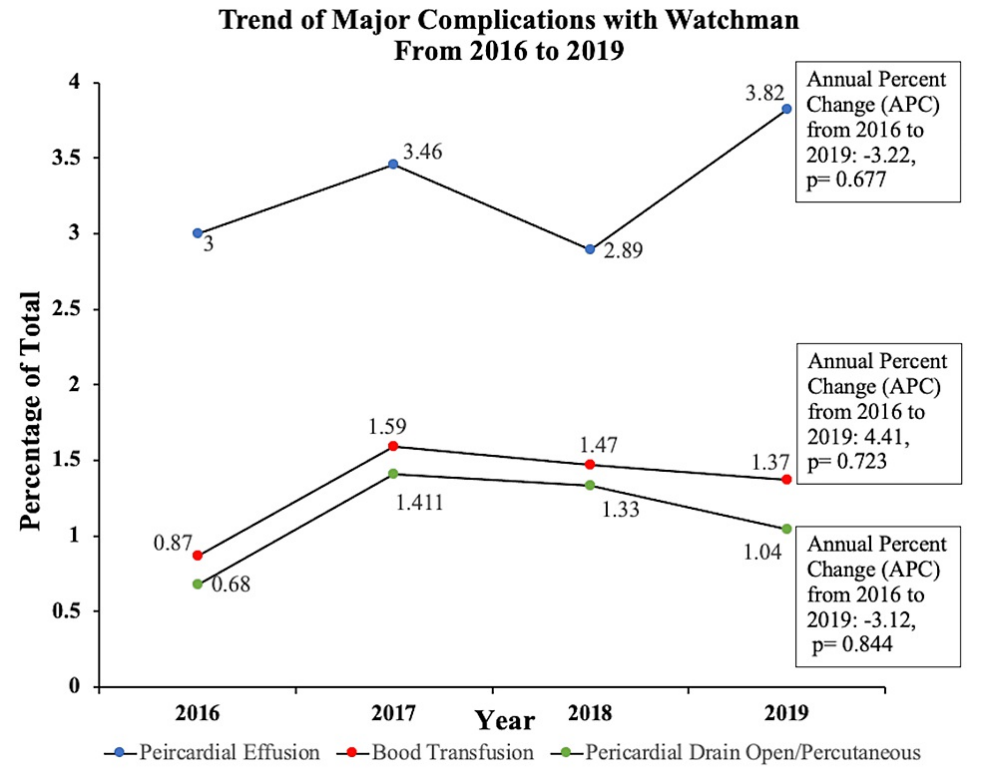
Diagram showing the yearly trend of Watchman Complication including pericardial effusion, significant bleeding requiring transfusion, and pericardial effusion requiring drain percutaneous/open from 2016 to 2019

Out of the weighted sample size (N) of 1,42,420,378 weighted population size (sample size (n) of 28,484,087), 1,891,020 hospitalized patients had atrial fibrillation/flutter in primary diagnosis, amongst which 60,350 had LAAO performed in the USA from 2016 to 2019 (Figure 1). The majority of the implants were performed in Caucasians (84.88%), males (58.40%), with a mean age of 76.12 years, at a teaching hospital (88.27%), in large bed size (66.34%), at an urban location (98.08%). The most common comorbidities noted were hypertension (86.88%), coronary artery disease (49.72%), diabetes (34.61%), and heart failure (34.03%). About 89.96% of the procedures were covered by federal insurance. The majority of the patients were discharged home (92.80%) after the procedure, with about 7.20 percent of the patient requiring disposition to other healthcare facilities. The mean cost of hospitalization was $26,016 per procedure. The average length of stay was 1.32 ± 0.015 days (Table 1). We noted a rapid increase in yearly utilization of Watchman from 1.12% in 2016 to 5.45% in 2019, with an annual percentage change (APC) of 62.30 and a p-value of 0.013 (Figure 2). Our complication rates were around 5.72%, with no significant change from 2016 to 2019 (APC: 6.23; p-value: 0.170) (Figure 2,3) 

**Table 1:** Baseline characteristics of the study population COPD: chronic obstructive pulmonary disease; CKD: chronic kidney disease; CABG: coronary artery bypass grafting; TIA: transient ischaemic attack 1 Comorbidities were coded using appropriate ICD-10 in the secondary diagnosis field as per supplemental material in appendix Table 3. 2 Quartile classification of estimated median household income of residents in the patient's ZIP code (demographic data obtained from Claritas) with the quartile identified by values 1 to 4, indicating the poorest to wealthiest populations. These are estimates, updated and early, and the Valley range can vary by year. 3 Federal insurance was defiant if primary payer were either Medicare or Medicaid. All other categories were defined as private insurance. 4 Bed size cut off are divided into small, medium, and large based on hospital beds and are specific to the hospital's location and teaching status. 5 Teaching hospital is considered to be a hospital if it is a member of the Council of Teaching Hospitals (COTH), has an AMA-approved residency program, or has a ratio of full time given it interns and residents to beds of 0.25 or higher. 6 CHA2DS2-VASc scores were calculated using appropriate ICD-10 in the secondary diagnosis field as per supplemental material in appendix Table 5. 7 Elixhauser Comorbidity Software Refined for ICD-10-CM 2021, Agency for Healthcare Research and Quality R, Rockville, MD.

Baseline characteristics	Overall (%) (N= 60,350)	No complication (%) (n=56,895)	Any complication (%) (n=3,455)	p-value
Watchman device implantation				
Age (years)				0.1
18-49	0.46	0.47	0.14	
50-64	6.7	6.74	6.08	
65-74	31.72	31.9	28.65	
>= 75	61.13	60.88	65.12	
Mean age	76.12	76.07	76.92	0.006
Gender				<0.001
Male	58.4	58.93	49.64	
Female	41.6	41.07	50.36	
Race				0.59
White	84.88	84.93	84.08	
Non-white	15.12	15.07	15.92	
Comorbidity^1^				
Obesity	17.3	17.2	18.96	0.25
Hypertension	86.88	86.89	86.83	0.97
Diabetes	34.61	34.62	34.44	0.92
Heart failure	34.03	33.68	39.94	0.001
Coronary artery disease	49.72	49.77	48.91	0.65
COPD	17.36	17.22	19.54	0.12
CKD stage 3 or more	15.18	14.86	20.41	<0.001
Prior CABG	27.73	28.15	21.13	0.001
Hyperthyroidism	0.09	0.08	0.29	0.07
Alcohol use disorder	1.41	1.45	0.72	0.11
Mitral valve stenosis	0.22	0.21	0.43	0.24
Prior stroke/TIA	27.1	27.2	25.47	0.32
Peripheral vascular disease	10.12	10.04	11.58	0.17
Anemia	18.11	16.87	38.64	<0.001
Median household income category for patient's Zip Code^2^				0.14
0-25 percentile	21.59	21.58	21.91	
26-50 percentile	25.83	26.06	22.06	
51-75 percentile	27.95	27.86	29.41	
76-100 percentile	24.63	24.51	26.62	
Primary payer^3^				0.73
Federal insurance	89.96	89.98	89.58	
Private insurance	10.04	10.02	10.42	
Hospital characteristics				
Hospital region				0.3
Northeast	16.59	16.46	18.67	
Midwest	22.42	22.33	23.88	
South	39.08	39.16	37.77	
West	22.09	22.05	19.68	
Hospital bed size^4^				0.07
Small	10.6	10.78	7.67	
Medium	23.06	23	24.02	
Large	66.34	66.22	68.31	
Hospital teaching status^5^				0.11
Non-teaching	11.73	11.6	13.89	
Teaching	88.27	88.4	86.11	
Hospital location				0.12
Rural	1.92	1.85	3.04	
Urban	98.08	98.15	96.96	
Disposition				<0.001
Home	92.8	93.82	75.98	
Facility/others	7.2	6.18	24.02	
In-hospital mortality	0.15			
Length of stay (mean ± SD) (days)	1.326 ± 0.015	1.219351 ± 0.013	3.09696 ± 0.149	<0.001
Cost of care (mean ± SD) (USD)	26016.72 ± 285.416	25601.75 ± 286.134	32887.87 ± 751.513	<0.001
CHA2DS2-VASc (mean; 95% confidence interval)^6^	3.45 (3.42 - 3.48)	3.44 (3.40 - 3.48)	3.58 (3.47 - 3.69)	0.015
Elixhauser (mean; 95% confidence interval)^7^	-0.66 (-0.76 to -0.56)	-0.70(-0.80 to -0.60)	-0.09 (-0.57 to 0.38)	0.01

The overall peri-procedural complication rate was 5.72%, and the in-hospital mortality rate was 0.15%. The most common complication noted was pericardial effusion in about 3.41%, followed by bleeding requiring transfusion in about 1.40%. Finally, pericardial effusion requiring drainage was noted in 1.16% (Table 2). Overall, 0.67% of the population required a repeat procedure/revision surgery. Stroke was noted in about 0.12% of the people, predominantly hemorrhagic stroke in about 0.07 %. Systemic embolism occurred in about 0.16 % of the population (Table 2). There was no difference in the complication rates by hospital region, bed size, teaching status, hospital location, or insurance status. Patients with comorbidities like CHF, CKD stage 3 or more, CHA2DS2-VASc score, and anemia were noted to have a higher prevalence of complications. The hospitalized patient who had complications post watchman had a higher mean Elixhauser comorbidity index than those who had no complications (-0.092 versus -0.699, p-0.01), with higher scores predicting a higher likelihood of adverse events (Table 1). Pericardial effusion occurs in 3.14% with no change in rates from 2016 to 2019 (APC: -3.22; p-value 0.677). We also noted the open/percutaneous drain requirement to be around 1.16%, with no change in rates from 2016 to 2019 (APC: -3.12; p-value: 0.844).

**Table 2:** Complications post-Watchman CHF: congestive heart failure 1 Comorbidities were coded using appropriate ICD-10 In the secondary diagnosis field as per supplemental material in appendix Table 4

Complications post-Watchman	%
Watchman device closure	60,350
Complications^1^	
Overall complications	5.72
Death	0.15
Total pericardial effusion	3.41
Pericardial drainage open/percutaneous	1.16
Requiring repeat procedure	0.67
Removal of intraluminal device	0.58
Endoscopic occlusion of left atrial appendage	0.01
Percutaneous occlusion of left atrial appendage other than Watchman	0.01
Open occlusion of left atrial appendage	0.07
Systemic embolism	0.16
Embolism due to cardiac and vascular prosthetic devices, implants, and grafts	0.02
Arterial thromboembolism	0.14
Implant displacement	0.07
Implant leak	0.01
Major bleeding requiring blood transfusion	1.40
Accidental puncture and laceration of a circulatory system organ or structure during a procedure	0
Post-procedure CHF	0.01
Cardiac arrest	0.03
Post-op hypotension	0.46
Total stroke	0.12
Post/intraprocedural cerebrovascular infarction following cardiac surgery	0.04
Hemorrhagic stroke	0.07
Ischemic stroke	0
Pneumothorax	0
Anesthesia complication	0
Air embolism	0

## Discussion

We utilized the large nationally representative database of inpatient hospitalization to identify the safety of watchman implantation in clinical practice in the USA. The Watchman device was the only FDA-approved device from 2016 to 2019 [3-5] for our study purpose. When compared with the previous trial study, including Protect AF, CAP Registry, PREVAIL trial, and EWOLUTION, we found that demographics of the patients who got Watchman device were older (76.12 years versus when compared to 71.7 to 74 years), males 58.40% (when compared to 59.9 to 70.4%) and had similar mean CHA2DS2-VASc score of 3.45 (when compared to 3.4 to 4.5 in previous studies) [6-8]. We also found a similar demographic of comorbidities, where 86.88% were hypertensive (when compared to 81.7% to 89.6%), 34.61% had diabetes (when compared to 24.4 to 33.8%), 27.10% had a prior history of stroke/TIA (when compared to 17.7 to 29.7 %), and 34.03% had a history of congestive heart failure (CHF) (when compared to 26.8 to 34.2%) [6-8]. Based on the above demographics, the mean age of the population is shifting towards the older female population compared to those recorded in previous trials.

The initial RCT for the Watchman device was the Protect-AF trial [6] which showed that immediate complication rates post-procedure were 8.7%. This was followed by CAP Registry and PREVAIL trial, which showed a lower complication rate of 4.2% and 4.5%, respectively [6-7]. EWOLUTION is the more recent cohort on the device’s safety profile in routine clinical practice reported complication rates of around 1.8% [8]. It is thought that this decreasing rate of compilation is because of maturing experience of implanting physicians. Other previous studies using the NIS database to see the adverse outcome of LAAO in the USA between 2006 and 2010 showed an overall higher complication rate (24.3%) compared with all of the studies [12]. However, this study was limited due to not explicitly assessing the complication rate associated with the Watchman device alone due to a single ICD 9 code for any type of LAAO, including epicardial and endocardial approaches. This was followed by using the NIS database of 2016 showed a complication rate of 1.9%. However, the study did not include all the complications seen in the EWOLUTION and Protect AF trial and had a smaller population size (N=5175) [13]. The complication rates found in our study were around 5.72%, with no significant change from 2016 to 2019 (APC: 6.23; p-value: 0.170). This was despite an increasing yearly utilization rate of Watchman from 1.12% in 2016 to 5.45% in 2019, with an annual percentage change (APC) of 62.30 and a p-value of 0.013. Pericardial effusion has been reported to range from 4.80% to 0.38% compared to our observation of 3.14%, with no change in rates from 2016 to 2019 (APC: -3.22; p-value 0.677). We also noted the open/percutaneous drain requirement to be around 1.16%, with no change in rates from 2016 to 2019 (APC: -3.12; p-value: 0.844). In our study, about 1.40% of watchman patients had significant bleeding requiring blood transfusion with no substantial change from 2016 to 2019 (APC: 0.723; p-value: 0.723) (Figure 2,3). Our mortality rate of 0.15% was lower than observed in the EWOLUTION and protect AF trial [7-8].

The most frequent complications noted in our study were hemorrhage and pericardial effusion, accounting for around 84 percent of the total complications. This may be because complications now are independent of operator use/technique but more dependent on the type of device used, as evident by lesser complication rates of 0.5% noted with newer devices like Watchman FLX, which had design changes like reduced metal exposure, more significant number of struts, distal tines were folded back and had more extensive size range with shorter device size [14]. Moreover, this may also be because the number of interventional cardiologists is increasing yearly, as evident by the number of interventional cardiologists rising from 3255 in 2015 to 4407 in 2019 [15]. Since these procedures are a learning curve that takes time to mature, the result from the NIS database may be more of an equilibration between expert interventionalist with developing ones. We also noted that complications were more common in the elderly female of the non-white race with a history of heart failure, prior coronary artery bypass graft, and CKD stage 3. In addition, the length of stay and hospital cost was more in the complication group. We also noted CHA2DS2-VASc, and Elixhauser Comorbidity scores were higher in the complication group when compared to the no-complication group (3.58 vs. 3.44; p-value: 0.01 and -0.092 versus -0.699, p-0.01, respectively), showing that the population who got complications had more comorbidity, to begin with, which may be contributing towards a higher probability of complications. 

This is the most extensive report available yet that helps us provide real-world national experience on peri-procedural adverse events of Watchman implantation. HCUP data is large and validated enough that the differences observed are likely clinically relevant. However, we acknowledge several limitations, some of which are inherent to data analysis. We used the best practices to find all the study population and complications. However, there might have been coding errors, unmeasured confounder, and under-reporting of comorbidities which are potential limitations of using ICD 10. In addition, we cannot assess challenges during the implantation, actual procedural experience, and the results of the device deployment. Complications that happened after the discharge cannot be accounted for. We also cannot quantify the experience of operating providers/institutions from the database, which is a significant contributor towards complications. 

## Conclusions

Our study demonstrates that the trend of complications with Watchman device implant didn’t change despite increased utilization. This might be due to characteristics inherent to the device and patient population. 

## Appendices

**Table 3:** ICD 10 coding for complications CHF: congestive heart failure

Complications	ICD 10 codes
Ischemic stroke	I63, G46
Hemorrhagic stroke	I61, I629
Requiring repeat procedure	
Removal of intraluminal device	"02PA0D", "02PA0DZ", "02PA3D", "02PA3DZ", "02PAXD", "02PAXDZ"
Endoscopic occlusion of left atrial appendage	"02L74", "02L74C", "02L74CK", "02L74D", "02L74DK", "02L74Z", "02L74ZK"
Percutaneous occlusion of left atrial appendage other than watchman	"02L73", "02L73C", "02L73CK", "02L73Z", "02L73ZK"
Open occlusion of left atrial appendage	"02L70C", "02L70CK", "02L70D", "02L70DK", "02L70Z", "02L70ZK"
Systemic embolism	I74, K550, N280, H340, H341
Draining pericardial cavity-open/percutaneous	0W9D
Total pericardial effusion	I31.4, I31.2, I31.3
Implant displacement	"T82528", "T82528A", "T82528D", "T82528S", "T82529", "T82529A", "T82529D", "T82529S"
Implant leak	"T82538", "T82538A", "T82538D", "T82538S", "T82539", "T82539A", "T82539D", "T82539S"
Implant infection	"T827", "T827XXA", "T827XXD", "T827XXS”
Accidental puncture and laceration of a circulatory system organ or structure during a procedure	"I9751"
Post-procedure CHF	"I97130", "I97131"
Post-procedure cardiac arrest	"I97120", "I97121", "I9712"
Post-op hypotension	"I9581"
Pneumothorax	“J93”
Anesthesia complication	"T88”
Air embolism	"T790XXD", "T790XXS", "T800", "T800XXA", "T800XXD", "T800XXS"
Intra/post-operative stroke	"I97820", "I97810", "I978", "I9782"
Hemorrhagic stroke	“I61”
Major bleed needing transfusion	Major Bleeding + Transfusion
Major bleeding	“I61”, “I62”, “I69”,“K92”, “I92”, “I85”, “K22”, “K25”, “K29”, “K26”, “K27”, “K57”, “K51”, “K50”, “K62”
Transfusion	"30233H", "30233H0", "30233H1", 30233N", "30233N0","30233N1", "30233P", 30233P0", "30233P1", "30243N", “30243N0", "30243P", "30243N1", "30243P0", "30243P1", "30243H", "30243H0", “30243H1"

**Table 4:** ICD 10 codes for co-morbidities COPD: chronic obstructive pulmonary disease; CKD: chronic kidney disease; CABG: coronary artery bypass grafting; TIA: transient ischaemic attack 1 Elixhauser Comorbidity Software Refined for ICD-10-CM 2021, Agency for Healthcare Research and Quality R, Rockville, MD. 2 NIS description of data elements

Co-morbidities	ICD-10 codes in secondary field
Obese^1^	Utilized Elixhauser mapping program to find all obesity cases
Hypertension^1^	Utilized Elixhauser mapping program to find all hypertension cases
Diabetes^1^	Utilized Elixhauser mapping program to find all diabetes cases
Heart failure^2^	I50, I09.81, I97.13, I11.0, I13.0, I13.2
Coronary artery disease^2^	"I25", "I25.2", "I25.2", "I25.6"
COPD^2^	J41, J42, J43, J44
CKD stage 3 or more^2^	"N183", "N1830", "N1831", "N1832", "N184", "N185", "N186"
History of CABG^2^	I25.7, I25.8, I25.9, Z98.61 Z95.5, T82.2, Z95.1
Hyperthyroidism^2^	E05
Alcohol disorder^1^	Utilized Elixhauser mapping program to find all alcohol use disorder cases
Mitral valve stenosis^2^	I34.2, I05.0/2,
Prior Stroke/TIA^2^	I69.3, Z86.73
Peripheral vascular disease^1^	Utilized Elixhauser mapping program to find all peripheral vascular disease cases
Anemia^2^	D50, D51, D52, D53, D55, D57, D58, D59, D60, D61, D62, D63, D64, D46, O99.0

**Table 5:** ICD 10 coding (in secondary diagnosis field) for CHA2DS2-VASc score TIA:  transient ischaemic attack 1 Elixhauser Comorbidity Software Refined for ICD-10-CM 2021, Agency for Healthcare Research and Quality R, Rockville, MD.

CHA_2_DS_2_-VASc Score variables	ICD-10 codes
Heart failure - 1 point	I50, I09.81, I97.13, I11.0, I13.0, I13.2
Hypertension^1 -^ 1 point	Utilized Elixhauser mapping program to find all hypertension cases
Age < 65 years - 0 point	Age variables is provided in NIS database
Age >=65, <=75 years - 1 point	
Age > 75 years - 2 points	
Diabetes^1^ - 1 point	Utilized Elixhauser mapping program to find all diabetes cases
Vascular disease (coronary, aortic or any peripheral vascular disease) - 1 point	I20, I21, I22, I23, I24, I25, T82.21, Z95.1, Z98.61, Z95.5, E08.5, E09.5, E10.5, E11.5, E13.5, I73, T82, Z98.62, Z95.820, I70, I71, I69, Z86,
Sex- male - 0 point	Sex information was provided in NIS database
Sex- female - 1 point	
History of TIA/stroke - 2 points	I69.3, Z86.73
